# A randomised controlled trial of therapist-assisted online psychological therapies for posttraumatic stress disorder (STOP-PTSD): trial protocol

**DOI:** 10.1186/s13063-020-4176-8

**Published:** 2020-04-23

**Authors:** Anke Ehlers, Jennifer Wild, Emma Warnock-Parkes, Nick Grey, Hannah Murray, Alice Kerr, Alexander Rozental, Esther T. Beierl, Apostolos Tsiachristas, Rafael Perera-Salazar, Gerhard Andersson, David M. Clark

**Affiliations:** 1 Department of Experimental Psychology, University of Oxford, Paradise Square, Oxford, OX1 1TW UK; 2 Oxford Health NHS Foundation Trust, Oxford, OX3 7JX UK; 3 Department of Psychology, Institute of Psychiatry, Psychology and Neuroscience, King’s College London, Denmark Hill, London, SE5 8AF UK; 4 South London and Maudsley NHS Foundation Trust, London, SE5 8AZ UK; 5 Sussex Partnership NHS Foundation Trust, Aldrington House, 35 New Church Road, Hove, BN3 4AG UK; 6 Centre for Psychiatry Research, Department of Clinical Neuroscience, Karolinska Institutet, Norra Stationsgatan 69, 113 64 Stockholm, Sweden; 7 Nuffield Department of Population Health, University of Oxford, Richard Doll Building, Old Road Campus, Oxford, OX3 7LF UK; 8 Nuffield Department of Primary Care Health Sciences, Radcliffe Observatory Quarter, University of Oxford, Woodstock Road, Oxford, OX2 6GG UK; 9 Department of Behavioural Sciences and Learning, Linköping University, 581 83 Linköping, Sweden

**Keywords:** Posttraumatic stress disorder, Randomised controlled trial, Clinical trial, Cognitive behaviour therapy, Cognitive therapy, Stress management, Trauma-focus, Digital intervention, Internet, Protocol

## Abstract

**Background:**

Over the last few decades, effective psychological treatments for posttraumatic stress disorder **(**PTSD**)** have been developed, but many patients are currently unable to access these treatments. There is initial evidence that therapist-assisted internet-based psychological treatments are effective for PTSD and may help increase access, but it remains unclear which of these treatments work best and are most acceptable to patients. This randomised controlled trial will compare a trauma-focussed and a nontrauma-focussed therapist-assisted cognitive behavioural Internet treatment for PTSD: Internet-delivered cognitive therapy for PTSD (iCT-PTSD) and internet-delivered stress management therapy (iStress-PTSD).

**Methods/design:**

The study is a single-blind, randomised controlled trial comparing iCT-PTSD, iStress-PTSD and a 13-week wait-list condition, with an embedded process study. Assessors of treatment outcome will be blinded to trial arm. Two hundred and seventeen participants who meet *DSM-5* criteria for PTSD will be randomly allocated by a computer programme to iCT-PTSD, iStress-PTSD or wait-list at a 3:3:1 ratio. The primary assessment point is at 13 weeks, and further assessments are taken at 6, 26, 39 and 65 weeks. The primary outcome measure is the severity of PTSD symptoms as measured by the PTSD Checklist for *DSM-5* (PCL-5). Secondary measures of PTSD symptoms are the Clinician Administered PTSD Scale for DSM-5 (CAPS-5) and the Impact of Event Scale-Revised (IES-R). Other symptoms and well-being will be assessed with the Patient Health Questionnaire (PHQ-9), Generalised Anxiety Disorder Scale (GAD-7), WHO (Five) Well-Being Index, Work and Social Adjustment Scale (WSAS), Endicott Quality of Life Scale (QoL), and Insomnia Sleep Index (ISI). Health economics analyses will consider quality of life, productivity, health resource utilisation, employment status and state benefits, and treatment delivery costs. Process analyses will investigate candidate mediators and moderators of outcome. Patient experience will be assessed by interview and questionnaire.

**Discussion:**

This study will be the first to compare the efficacy of a trauma-focussed and nontrauma-focussed therapist-assisted online cognitive behavioural treatment for people with posttraumatic stress disorder.

**Trial registration:**

ISRCTN16806208. Registered prospectively on 5 January 2018.

## Background

Posttraumatic stress disorder (PTSD) is a common and disabling stress disorder with an estimated 12-month prevalence of 1.3 to 3.6% [1]. Over the last two decades, significant advances in the understanding and treatment of PTSD have been made. Several versions of cognitive behavioural therapies have been shown to be effective. The UK National Institute for Health and Care Excellence (NICE) [2] and international treatment guidelines [3–5] currently recommend trauma-focussed psychological therapies, including trauma-focussed cognitive behavioural therapy (TF-CBT) and eye-movement desensitisation and reprocessing (EMDR), as first-line treatments for PTSD. A recent Cochrane review [6] of psychological therapies for PTSD, however, suggested that nontrauma-focussed cognitive behavioural therapies may achieve similar outcomes (at least in the short term), and, like TF-CBT, are superior to wait-list and other therapies. Further direct comparisons to evaluate the relative merits of trauma-focussed (such as cognitive therapy for PTSD [7]) and nontrauma-focussed cognitive behavioural therapies (such as stress-management therapy [8]) in the treatment of PTSD are warranted.

Despite advances in treatment options, many people with PTSD are currently unable to access effective psychological treatments due to a range of factors, such as shortage of therapists, living too far away from treatment centres, mobility problems, or being unable to attend therapy during usual working hours due to work or childcare. Given the large number of people suffering from PTSD, it is, therefore, desirable to develop more efficient forms of treatment delivery that can be widely accessed, and online treatment delivery appears to be a promising alternative to face-to-face therapy. There is initial evidence that therapist-assisted, Internet-based psychological treatments are effective for PTSD [8–13]. In a meta-analysis based on 11 trials and 1139 participants, Sijbrandij et al*.* [14] reported an average between-group effect size at post treatment of *d* = 0.71 compared to wait-lists or treatment as usual. It remains unclear which of these internet-based treatments work best and are most acceptable to patients, and whether they are cost-effective [15, 16].

The present study will compare a novel, trauma-focussed therapist-assisted online psychological therapy (internet-based cognitive therapy for PTSD, iCT-PTSD) and a comprehensive nontrauma-focussed therapist-assisted online psychological therapy (internet-based stress management therapy, iStress-PTSD). Both treatments will be compared with a wait-list to control for the natural recovery that is sometimes seen in PTSD samples.

Cognitive therapy for PTSD is a TF-CBT programme that has been shown to be highly effective and acceptable to patients [7, 17–20]. The treatment focusses on changing problematic appraisals that induce a sense of current threat, updating trauma memories and changing problematic behaviours that maintain the problem. A pilot study of an internet-delivered version of this treatment suggested that it may be as effective as face-to-face CT-PTSD [9].

Nontrauma-focussed CBT programmes for PTSD focus on teaching strategies that help patients cope better with PTSD symptoms, solve problems and reduce avoidance. The present study will use internet-based stress management therapy (iStress-PTSD), developed by Andersson and colleagues, a very comprehensive CBT stress-management programme that includes applied relaxation, mindfulness, thought challenging, and exposure to avoided situations. It has been shown to be effective in several randomised trials [8, 10]. The programme was translated into English and adapted for patients with PTSD.

The primary objective of the trial is to determine

1. Whether iCT-PTSD is superior to iStress-PTSD in reducing symptoms of PTSD

Other objectives are to determine

2. Whether iCT-PTSD and iStress-PTSD are efficacious, i.e. whether they lead to greater improvement in PTSD symptoms than a wait-list condition
3. Whether iCT-PTSD leads to greater improvement in depression, anxiety, well-being, disability, quality of life and sleep problems than iStress-PTSD
4. Whether iCT-PTSD and iStress-PTSD lead to greater improvement in depression, anxiety, well-being, disability, quality of life and sleep problems than a wait-list condition
5. Whether iCT-PTSD is cost-effective compared with iStress-PTSD in terms of cost per participants with a clinical improvement in PTSD symptoms and costs per quality-adjusted life year (QALY) gained

## Methods/design

### Design

The design is a single-blind, randomised controlled trial comparing two therapist-assisted internet-based psychological treatments for PTSD and a wait-list condition (superiority trial), with an embedded process study. Assessors of treatment outcome will be blinded.

### Study setting

The study will be conducted in three locations in the UK (Oxford, London, and Brighton and Hove). Participants will be recruited from primary care Improving Access to Psychological Therapies (IAPT) services in rural and urban areas (Buckinghamshire, Berkshire, Croydon, Lambeth, Lewisham, Oxfordshire, Southwark, Brighton and Hove, and East Sussex). Self-referrals and referrals from other National Health Service (NHS) services are also accepted.

### Eligibility criteria

Table 1 shows the inclusion and exclusion criteria. Eligibility criteria were chosen to recruit a representative sample of patients with PTSD treated in IAPT services in the UK with a wide range of PTSD severity. Comorbidities such as comorbid depression, other anxiety disorders, or substance misuse and a history of previous trauma, including childhood abuse, and previous treatment for PTSD are common in these patients and will *not* be used as exclusion criteria.

**Table 1:** Therapist-assisted, online psychological therapies for posttraumatic stress disorder (STOP-PTSD) inclusion and exclusion criteria

Inclusion criteria	
Participants must meet the following criteria:	
1. Aged 18 years and above	
2. Willing and able to provide informed consent	
Meet the diagnostic criteria for PTSD as determined by the Structured Clinical Interview for *the Diagnostic and Statistical Manual of Mental Disorders, Fifth Edition* (*DSM-5*) [21]	
3. Their current reexperiencing symptoms are linked to one or two discrete traumatic events that they experienced in adulthood or adolescence, or several traumatic episodes during a longer period of high threat (e.g. domestic abuse, war zone experiences)	
4. PTSD is the main psychological problem needing treatment	
5. Able to read and write in English	
6. Access to the Internet	
7. Willing to be randomly allocated to one of the psychological treatments or wait-list	
8. If taking psychotropic medication, the dose must be stable for at least 1 month before randomisation	
9. If currently receiving psychological therapy for PTSD, this treatment must have ended before randomisation	
Exclusion criteria	
A person is not eligible if any of the following apply (assessed by clinician in the initial clinical assessment);	
1. History of psychosis	
2. Current substance dependence	
3. Current borderline personality disorder	
4. Acute serious suicide risk	

**Fig. 1:**
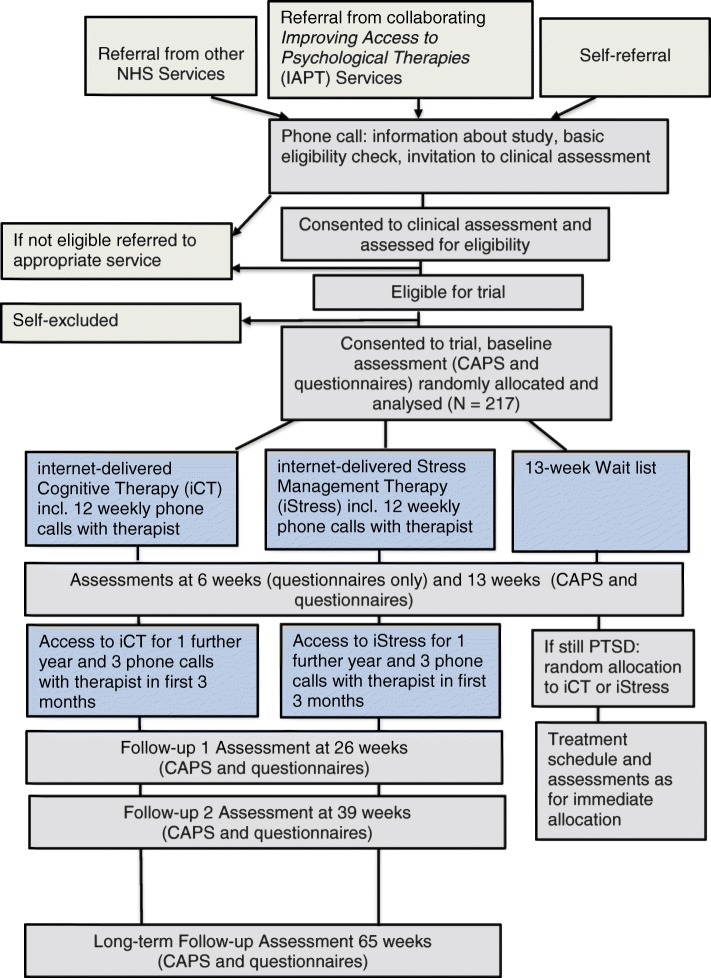
Participant time line of the activities during the trial

**Fig. 2:**
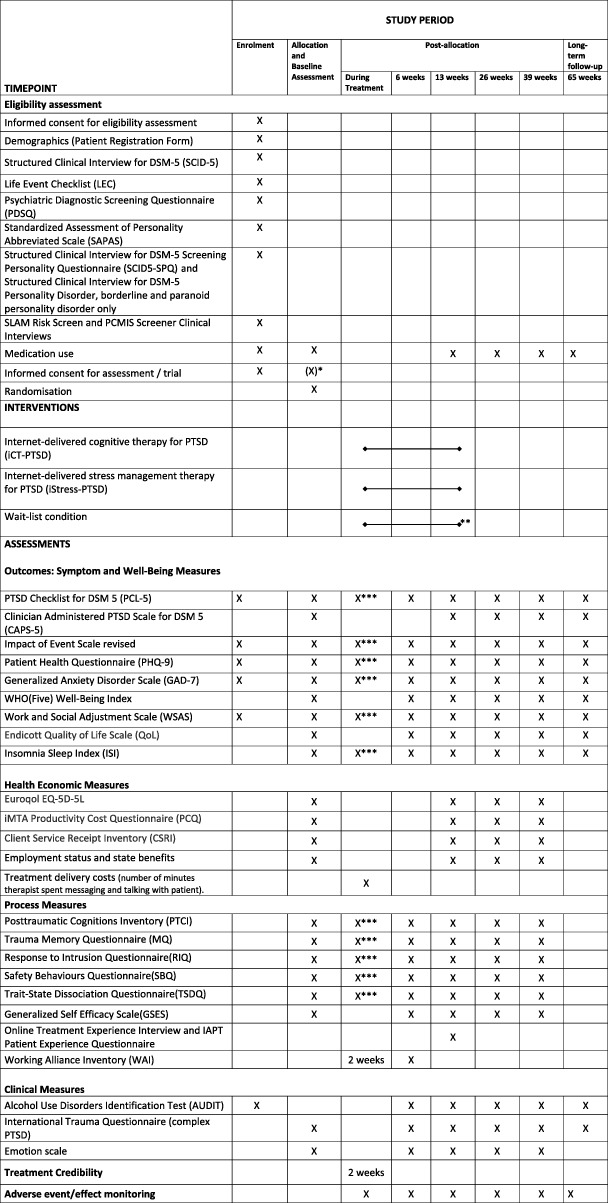
Schedule for enrolment, interventions and assessments. *If Informed Consent Form **(**ICF) part 2 is not completed at the time of the eligibility assessment it will be completed at the beginning of the baseline visit ahead of randomisation. **Followed by random allocation to iCT-PTSD or iStress if still meets criteria for posttraumatic stress disorder (PTSD). ***Collected by the online therapy programme, Improving Access to Psychological Therapies Services require weekly measures of IES-R, PHQ-9, GAD-7 and WSAS for patient records

Figure 1 shows the participant time line of activities during the trial**.** The methods of enrolment, interventions and assessments are summarised in Fig. 2. Potential participants who are referred by the collaborating IAPT services, other NHS services, or who self-refer, will receive information about the trial on the phone, answer basic questions about eligibility (e.g. age, access to the internet), will be sent an information sheet and will have the possibility to ask questions. The information sheet provides information about the aims of the study, the clinical assessment and random allocation process, treatment and assessment schedule with details of time involved (including a flowchart), potential benefits and risks of taking part, ethical approval, sponsor and funder information, data management and confidentiality, freedom to withdraw at any time, financial reimbursement and contact details. If they are interested, potential participants will be invited for a clinical eligibility assessment by a clinical psychologist at one of the trial locations. Participants will be given written consent for the assessment, which will include the Life Event Checklist for *DSM-5* (LEC-5) [22], PTSD Checklist for *DSM-5* (PCL-5) [23], Impact of Event Scale-Revised (IES-R) [24], Patient Health Questionnaire (PHQ-9) [25], Generalised Anxiety Disorder 7-item Scale (GAD-7) [26], Work and Social Adjustment Scale (WSAS) [27], Alcohol Use Disorders Identification Test (AUDIT) [28], Structured Clinical Interview for *DSM-5* [21] (for disorders screening positive on the Psychiatric Diagnostic Screening Questionnaire [29]), and the borderline and paranoid personality disorder section of the Structured Clinical Interview for *DSM-5* Personality Disorders [30] (if screening positive on the Structured Clinical Interview for *DSM-5* Screening Personality Questionnaire [31]), Standardised Assessment of Personality Abbreviated Scale (SAPAS) [32], and standardised risk assessments assessing suicide risk and risk to others, including ongoing threat from others.

If the clinical assessment shows that the participant is eligible for the trial, they will be informed, will have the opportunity to ask further questions and, if they agree to participate, sign the Informed Consent Form for the trial. Those who are not eligible or do not agree to participate in the trial will be advised about treatment options and referred to the appropriate services.

### Interventions

iCT-PTSD and iStress-PTSD will be delivered online via a series of therapy modules over 3 months, with the therapist support by messages within the programme, SMS and weekly phone calls (on average taking 4 h total of therapist time per participant). The therapist releases the modules gradually, two to three modules per week. Participants will retain access to the programme for a year post intervention and will be able to print and keep the therapy modules that they completed online. They will have up to three monthly phone calls with the therapist in the first 3 months of follow-up to help maintain treatment gains. Therapists will be experienced in cognitive behavioural therapy and will have received training in the delivery of both online treatments and have treated at least two supervised training cases in each condition.

The online treatments have the same user interface and design, and can be accessed by PCs, tablets or smart phones. Participants who do not have a PC or tablet will be able to borrow a tablet for the duration of treatment. Both interventions will be delivered via online modules that are identical in design and share the same multimedia features to facilitate engagement and accessibility (text with information and patient examples, videos of a therapist talking or whiteboard videos for key information, videos of patient testimonies, questions and text boxes for the patient to complete, audio-recordings for the patient to listen to, graphs and pictures). The modules and design were developed with extensive input from service-users. Therapists can, with the participant’s knowledge, read the information that the participant provides in the modules and can write notes for the participants directly into the modules.

#### iCT-PTSD

iCT-PTSD is the internet-delivered version of cognitive therapy for PTSD, one of the TF-CBT programmes recommended by NICE and international treatment guidelines [2, 3, 5]. The treatment modules (see Table 2) focus on changing problematic appraisals that induce a sense of current threat, updating trauma memories and changing problematic behaviours that maintain the problem. It does not include iStress-PTSD’s training in stress-reduction strategies (such as applied relaxation and mindfulness). An uncontrolled pilot study suggested that iCT-PTSD may be as effective as face-to-face CT-PTSD [9].

**Table 2:** Internet-delivered cognitive therapy for posttraumatic stress disorder (iCT-PTSD) modules

The following modules will be released to all participants assigned to iCT-PTSD, as they represent core procedures of CT-PTSD:	
1. Introducing the Treatment	
2. Reclaiming your Life (with my Weekly Plan)	
3. It’s All Understandable	
4. Updating your Memories – Part 1 – Telling the Story of your Trauma	
5. Updating your Memories – Part 2 – Finding your Hot Spots	
6. Updating your Memories – Part 3 – How to Update your Hot Spots	
7. Updating your Memories – Part 4 – Updating your Hot Spots	
8. Spotting Memory Triggers	
9. Beating Memory Triggers – Part 1 – THEN versus NOW	
10. Beating Memory Triggers – Part 2 – THEN versus NOW practise using the My Triggers page	
11. Beating Memory Triggers – Part 3 – Tackling Triggers in Everyday Life	
12. Understanding and Dealing with Risk – Part 1	
13. Understanding and Dealing with Risk – Part 2	
14. My Site Visit	
15. My Blueprint	
16. Preparing for your First Follow-up	
17. Preparing for your Second Follow-up	
18. Preparing for your Final Follow-up	
In addition, the therapist can release the following optional modules, depending on the individual case formulation:	
19. Rumination	
20. Overcoming Shame and Humiliation	
21. Dealing with Anger	
22. Dealing with Guilt	
23. Sleep	
24. Dissociation	
25. I am Physically Different Now	
26. Earlier Memories	
27. Childhood Trauma	
28. Self-esteem	
29. Chronic Pain and PTSD	
30. Death of a Loved One	
31. Panic Attacks	
32. Overcoming Depression	
33. Managing your Inner Critic	
34. Dealing with Drugs and Alcohol	

#### iStress-PTSD

The iStress stress management therapy programme developed by Andersson and colleagues has been shown to be effective in several trials [8, 10]. It was adapted for people with PTSD and includes psycho-education about PTSD, training in problem-solving and training in techniques for stress reduction and coping with PTSD symptoms such as applied relaxation training, challenging irrational thoughts, mindfulness and training to improve sleep efficiency, as well as exposure to avoided situations. Treatment modules are listed in Table 3. Patients also work on challenge areas of their choosing such as coping with memories or worry. Therapists will focus on supporting the participant in learning and practising the stress management skills. Participants will choose to which stressful situations they will apply the techniques. Some participants may apply a trauma focus to some tools, such as thought challenging and using exposure to overcome avoidance to trauma triggers, such as people, places or situations. If they choose to do this, therapists will support them with trauma-focussed applications of the stress management techniques, but therapists will not direct participants to adopt trauma-focussed applications of exposure and thought challenging if they do not choose to do so. The programme does not include iCT-PTSD’s specific trauma-focussed procedures for testing trauma-related appraisals or working on the content of trauma memories and their triggers.

**Table 3:** Internet-delivered stress management therapy (iStress-PTSD) modules

The following modules will be released to all participants assigned to iStress-PTSD:	
1. Introduction	
2. About Stress – Part 1	
3. About Stress – Part 2	
4. In Balance – Part 1	
5. In Balance – Part 2	
6. Challenge your Thoughts – Part 1	
7. Challenge your Thoughts – Part 2	
8. Sleep and Mindfulness – Part 1	
9. Sleep and Mindfulness – Part 2	
10. Overcoming Challenges	
11. Overcoming Challenges Continued	
12. Plan your Time	
13. Be Kind to your Brain	
14. Maintenance and Closure	
15. Preparing for your First Follow-up	
16. Preparing for your Second Follow-up	
17. Preparing for Final Follow-up	
18. For participants who dissociate, therapists release an additional module: Understanding Dissociation	
In addition, the participant completes a range of diaries (e.g. stress diary, relaxation diary, exposure diary) and chooses which of the following challenge areas connected to their PTSD they will work on (each of these is addressed in three to four modules):	
19. Anger (3 parts)	
20. Coping with Memories (3 parts)	
21. Drugs and Alcohol (4 parts)	
22. Pain (4 parts)	
23. Worry (4 parts)	

#### Wait-list

Participants allocated to the wait-list condition will receive assessments only at baseline, 6 weeks and 13 weeks, and will then be randomly allocated to iCT-PTSD or iStress if they still meet the criteria for PTSD. If they no longer have PTSD, their participation in the trial is finished.

#### Treatment fidelity and credibility

Therapists will receive weekly group supervision for each of the treatment conditions to ensure adherence to the protocol and quality of treatment delivery. The iCT-PTSD supervision group will be led by Professor Anke Ehlers, and the iStress-PTSD supervisor will be Dr. Alexander Rozental. Therapists’ adherence to treatment components will be assessed by independent raters from messages therapists send via the online system and randomly selected audio-recordings of the phone calls between therapist and participant. Competence of delivery will be assessed by an independent clinical psychologist, using rating forms informed by the Cognitive Therapy Rating Scale-Revised [33]. Participants will be made aware of this in the participant information sheet. Credibility of treatments will be assessed with the self-report Borkovec and Nau’s Credibility Scale [34] in week 2 of treatment.

### Outcomes

#### Measures of symptoms and well-being

Details of assessment instruments and time points are found in the Standard Protocol Items: Recommendations for Interventional Trials (SPIRIT) Checklist (Fig. 2). The primary assessment is at 13 weeks (end of weekly phone calls/wait-list).

The primary outcome measure is the PTSD Checklist for *DSM-5* (PCL-5) [23], a standardised self-report measure of PTSD symptoms according to the *DSM-5* which is expected to yield the most complete data. Further measures of PTSD symptom severity include the IES-R [24], which is the PTSD measure used by IAPT services, and the Clinician Administered PTSD Scale for *DSM-5* (CAPS-5) [35], a semi-structured clinician-administered diagnostic interview for PTSD symptoms. The latter will be conducted by an independent trained assessor (a psychologist who is not one of the trial therapists) who will be unaware of the participant’s trial arm.

Measures of other symptoms and well-being include the Patient Health Questionnaire (PHQ-9) [25] to assess symptoms of depression; the Generalised Anxiety Disorder Scale 7-items (GAD-7) [26] to assess symptoms of anxiety; the WHO(Five) Well-Being Index [36] to assess psychological well-being; the Work and Social Adjustment Scale (WSAS) [27] to assess disability and interference with functioning; the Endicott Quality of Life Scale (QoL) [37] to assess quality of life and the Insomnia Sleep Index (ISI) [38] to assess sleep disturbance.

#### Measures for health economics analysis

The health economics analysis will use a measure of resource use, the Client Service Receipt Inventory (CSRI) [39]; the iMTA Productivity Cost Questionnaire (PCQ) [40]; two measures of quality of life, the EuroQol-5 Dimensions-5 Levels health survey (EuroQol EQ-5D-5 L) [41] (which is commonly used in health economic analyses but may be less sensitive to the effects of psychological interventions as most items refer to physical health) and the Endicott Quality of Life Scale (QoL) [37]; as well as employment status and state benefits. Treatment delivery costs will be calculated from therapist records of the number of minutes per week that the therapist spend messaging and talking with the participant.

#### Process measures

Process measures for analyses of patterns of change during treatment and mediation effects include short versions of the Posttraumatic Cognitions Inventory (PTCI) [42], Trauma Memory Questionnaire (MQ) [43], Response to Intrusion Questionnaire (RIQ) [44, 45], Safety Behaviours Questionnaire (SBQ) [46], Trait-State Dissociation Questionnaire (TSDQ) [45], and Generalised Self Efficacy Scale (GSES) [47]. In addition, participants and therapists complete the Working Alliance Inventory (WAI) [48], at 2 and 6 weeks. The Online Treatment Experience Interview and IAPT Patient Experience Questionnaire [49] will provide qualitative and quantitative information on patient treatment experience. Participants’ compliance with treatment will be assessed by the types and percentage of core modules completed, time spent on the programme, and weekly therapist ratings of module and assignment completion.

#### Additional measures

Participants will also complete some measures to help the therapist with selecting the appropriate module, for moderation analyses and collect descriptive information on the sample. These include the Alcohol Use Disorders Identification Test (AUDIT) [28], International Trauma Questionnaire [50] (to assess features of complex PTSD), and an emotion rating scale. The Patient Registration Form will collect demographic information.

### Sample size

The trial has been powered to detect an effect size of Cohen’s *d* = 0.50 for the comparison between iCT-PTSD and iStress-PTSD. This effect size was chosen as it corresponds to clinically meaningful differences on the primary outcome measure (PCL-5), and was used in the NICE [1] guidelines to define a clinically significant difference in placebo-controlled trials. For the comparison between psychological treatments and wait-lists an effect size of *d* = 0.80 is considered as clinically meaningful by NICE [1].

To detect a difference with an effect size of *d* = 0.50 with 80% power at *α* = .05, 63 participants per group are required. In addition, we have allowed for effects of clustering of observations within therapists, design factor = 1.18, assuming a conservative intra-class correlation of 0.01 (following Baldwin et al.’s recommendation [51]) and an average cluster size of 12 and a coefficient of variation of CV = .68, and conservatively allowed for 15% for drop-outs, yielding a sample size of 91 per group for this comparison [52]. Thus, as the initial allocation ratio is 3:3:1 (iCT-PTSD: iStress-PTSD: wait-list), 93 participants will be allocated to each of the treatment conditions, and 31 to the wait-list, and a total of 217 participants will be randomly allocated. The power to detect a difference between the treatments and wait-list conditions of *d* = 0.80 is greater than 98%.

### Allocation

After the eligibility assessment and giving informed consent for the trial, eligible participants will be randomised to one of the three trial conditions (iCT, iStress, wait-list) at a 3:3:1 ratio, stratified by location (Oxford, London, and Brighton and Hove), duration of PTSD (less than 18 months/18 months and above), and severity of PTSD symptoms on the PCL (high versus low), using an online random allocation programme developed by the Primary Care Clinical Trials Unit at the University of Oxford for this study. The programme uses a minimisation algorithm with a random component. The allocation sequence is not visible to the administrators who generate the treatment allocation with the programme.

For participants taking psychotropic medication, randomisation will take place after they have been on a stable dose for 1 month. For those currently receiving another psychological treatment, randomisation will take place after the end of this treatment. Participants originally allocated to the wait-list who have not recovered from PTSD at 13 weeks (end of wait) will be randomised to either iCT-PTSD or iStress at a 1:1 ratio.

### Blinding

Assessors of treatment outcome will be blinded. Therapists supporting the Internet-based treatments, trial administrators and participants will not be blind to treatment allocation due to the nature of the intervention.

### Recruitment

Recruitment will be mainly via referral from collaborating IAPT services (Buckinghamshire, Berkshire, Croydon, Lambeth, Lewisham, Oxfordshire, Southwark, Brighton and Hove, and East Sussex). Referrals from general practitioners (GPs) and other NHS services, such as Hospital Trauma Services or local therapists, are also accepted. Consent will be obtained for the GP or referring NHS service to be informed about the participants’ progress in therapy. Participants can also self-refer in response to information listed on the research team’s website (https://www.psy.ox.ac.uk/research/oxford-centre-for-anxiety-disorders-and-trauma/anxiety-disorders/post-traumatic-stress-disorder), trial registration websites (ISRCTN and UK Clinical Trials Gateway), or the University of Oxford’s trial website, email circulars at the collaborating site, and posters in GP practices, or media reports (such as local radio or newspapers) about the study.

### Data collection

Data will be collected from all participants, including those who discontinue treatment. Most of the outcome data will be collected via Qualtrics (anonymised by participant ID number), a widely used data collection software programme. If participants prefer, paper-and-pencil measures (anonymised by participant ID), these will be provided. Data for the process analyses will be captured online via the online therapy programmes (weekly measures). Access to the data will be restricted to named study personnel only and via a secure login (two-factor authentication).

The independent CAPS-5 assessors will receive comprehensive training in conducting and scoring the interview. Interrater reliability will be determined and discrepancies will be resolved by consensus.

In the event of source data being collected on paper, including the SCID-5 interview at the eligibility clinical assessment and the CAPS-5 interview at baseline (initial), 13, 26, 39 and 65 weeks, an exact copy of the anonymised data will be manually entered into the trial database by named study personnel, with the electronic data record being verified against the original paper record.

To aid retention, participants will be reimbursed £20 for completing each trial assessment at baseline, 6 weeks (including measures during treatment), 13 weeks, 26 weeks, 39 weeks and 65 weeks (see Fig. 2). They will not be paid for the clinical eligibility assessment and treatment.

Dropout, or premature termination from the study or treatment at any point after randomisation, will be recorded along with reason for discontinuation or termination. Participants can choose to withdraw from the trial intervention, withdraw from follow-up, withdraw from both aspects, or withdraw from both aspects and ask that previously collected data not be used. Their care in the NHS will not be affected at any time by declining to participate or withdrawing from the trial. A participant may be withdrawn from the intervention if they develop a condition which would exclude them from the study based on the eligibility criteria or a change in their clinical condition requiring urgent other treatment (e.g. the participant develops a psychotic disorder). Participants who withdraw/are withdrawn from the intervention part of the trial will continue in follow-up unless they withdraw their consent for this. Withdrawn participants will not be replaced.

Unless a participant has withdrawn consent to participation, repeated attempts using different approaches will be made to contact participants who cannot be easily contacted at assessment points. For any participant reluctant to complete the full outcome assessment at follow-up we will attempt to obtain the PCL-5, IES-R, PHQ-9, GAD-7, WSAS and ISI as a minimum dataset. As much information as possible will be collected from protocol non-adherers including reasons for non-adherence.

### Data management

The trial staff will ensure that the participants’ anonymity is maintained. The trial will comply with the Data Protection Act, which requires data to be anonymised as soon as it is practical to do so. Participants will be identified by a participant ID number on outcome measures and any electronic trial database. Personal information will be kept separate from the trial data.

No interim analyses are planned. For statistical analysis, the anonymised data will be downloaded onto password-protected computers. Any changes made to the data will be stored in the audit log with a full history of changes being recorded. The final data file of the anonymised data will be accessible to the trial statisticians, health economist and principal investigators (PIs). It will be maintained by the trial statisticians and stored on a secure Oxford University server for 10 years post completion of the trial.

The internet programme supporting the online therapies was developed in collaboration with FRY-IT (https://www.fry-it.com). It has numerous security features representing current best practice. It will employ secure client-server communication, full encryption of the server database, enforcement of strong passwords, two-factor authentication (i.e. login requires both a password and a PIN sent to the participant’s mobile phone) and hosting on a tier-4 hosting server. External access to the database using SSH protocol is prohibited. The system has been subjected to industry-standard penetration testing.

### Statistical methods

#### Analysis of symptom and well-being measures

All analyses will be intent-to-treat from randomisation. The primary outcome measure will be the PCL-5 and the primary assessment point will be 13 weeks (end of weekly treatment calls/wait). Group comparisons on mean scores of primary and secondary symptom outcome measures will be performed with linear mixed-effect regression models. Both the main effects of repeated assessments and condition and their interactions are specified, and baseline scores are included as a covariate. Superior outcome will show in significant treatment condition or time x treatment condition interactions. All randomised cases will be included in the analyses, irrespective of missing data (mixed models and structural equation models account for data missing at random). Missing data mechanisms will be explored and reported.

The effect size for the primary research question will be categorised as statistically significant, trend for superiority (nonsignificant effect size of *d* = 0.25 and above), possible small superiority (nonsignificant effect size between *d* = 0.10 and *d* < 0.25), possible equivalence (nonsignificant effect size between *d* < 0.10 and *d* > − 0.10). Analyses will explore effects of location and therapist on outcome. A range of potential covariates will be considered, for example: duration of PTSD, age at trauma, trauma type, number of traumas, severity of physical consequences of the trauma, history of childhood trauma, comorbid major depression, comorbid anxiety disorder, substance use, SAPAS score, gender, education level, ethnicity. A complier-adjusted causal effect analysis (CACE) [53] will account for differences in participant adherence to the protocol. Details will be specified in a separate statistical analysis plan.

#### Process evaluation

A process evaluation conducted alongside the main trial will explore moderators of outcome and mediators of change. Candidate moderators include comorbid depression, disturbances in self-organisation, substance use, education level, gender, treatment credibility. Mediators of treatment outcome to be considered are processes hypothesised to maintain PTSD (problematic appraisals, memory qualities, maintaining behaviours such as rumination and thought suppression) and self-efficacy. Details will be specified in a separate statistical analysis plan.

#### Health economic evaluation

A full economic evaluation will be performed to compare the costs and effects of providing iCT-PTSD versus iStress-PTSD. The results of the economic evaluation will be expressed in costs per patient with a clinical improvement in PTSD symptoms and costs per QALY gained, using non-parametric bootstrapping and Cost-Effectiveness Planes and Cost-Effectiveness Acceptability Curves. Details will be specified in a separate health economic analysis plan.

### Monitoring

There are no stopping rules for the trial as it is a low-risk trial that does not use Investigational Medicinal Products (non-CTIMP).

#### Trial monitoring

The independent Trial Oversight Committee (TOC) will review the protocol and the statistical analysis plan, and regularly review recruitment rates, compliance with protocol, adverse events and effects, and completeness of data collection at least annually. Members are independent experts specialising in clinical trials, PTSD, or statistics and a service-user representative. The Trial Management Committee will meet monthly to oversee trial procedures and progress.

#### Safety

Adverse effects or events will be monitored throughout treatment (during weekly phone calls with the therapist) and follow-up (during independent assessments) [54]. Number and type of adverse events will be reported in the main trial publication. All serious adverse events (SAEs) must be reported immediately (and within 24 h of knowledge of the event) to the investigator and will be reported to the Research Ethics Committee (REC) that gave a favourable opinion of the study if they are ‘related’ (resulted from administration of any of the research procedures) and ‘unexpected’ in relation to those procedures. In addition to the usual SAE categories, for the purposes of this trial severe self-harm and harm to others must be reported. Therapists will be asked to notify the PI directly should they be concerned at any time that a participant has caused, or is likely to cause, significant harm to themselves. The therapist should conduct a risk assessment and also inform the participant’s GP. Therapists will be asked to inform the appropriate authorities directly should they become concerned at any time that a participant has, or is likely to cause significant harm to others. Assessors will be asked to complete a brief risk assessment if the participant discloses suicidal ideation and should discuss with a member of the clinical team what action to take.

### Dissemination

The results of the trial will be published in peer-reviewed international journals. In line with Wellcome Trust guidance, publications will be made open access. In addition, information about the results will be made available to the participating services, NHS Trusts, participants, and to the wider public by media releases, public engagement events and University and NHS Trust websites.

## Discussion

This study will be the first to compare the efficacy of a trauma-focussed and nontrauma-focussed, therapist-assisted, online cognitive behavioural treatment for people with PTSD. The study will investigate whether these treatments are acceptable to patients and whether they are effective. The treatments will be compared on a broad range of outcomes in addition to PTSD symptom severity, including well-being and quality of life measures, patients’ experience of completing the online treatments and a health economic analysis. While we expect that both treatments will lead to substantial improvement, the results will be informative about their relative benefit across outcomes, and their potential in increasing access to cognitive behaviour therapy for PTSD.

Possible advantages of online treatments include convenience for patients in that they can work on the treatment in a place and at a time that suits them and do not need to travel to therapy. They may also worry less about possible stigma with an internet-based treatment. Another possible advantage is that the treatments are efficient as they require only 20 to 25% of the therapist time compared to face-to-face therapy. Thus, more patients can be treated in the same time. It is also possible that treatment fidelity may be more consistent across therapists than for face-to-face therapy, as the content of the treatments is mainly delivered through online modules.

Possible challenges include drop-outs from treatment and low compliance in completing the online modules. Previous studies of online treatments for psychological disorders have found high drop-out rates [55]. The development of iCT-PTSD and iStress-PTSD was facilitated by service-user involvement, which helped make the platform and modules user-friendly. Nevertheless, the results from this trial may help identify further ways to enhance treatment acceptability.

Another challenge is that while iStress-PTSD was chosen to represent a nontrauma-focussed treatment and focusses on general stress management skills to stressors in everyday life, it contains some trauma-related content. For example, the *About Stress* modules explain and normalise the effects of traumatic events as stress reactions, the *Coping with Memories* and *Understanding Dissociation* modules teach participants to apply the techniques to their intrusive memories, and all modules give examples of other people who experienced trauma and how the modules or tools helped them. Some participants may use skills such as thought challenging and exposure to trauma-related thoughts and situations. Thus, there will be a degree of overlap between the treatments in CBT techniques and trauma-related content despite their different focus. The degree of trauma-focus in both treatments will be monitored so that it can be included in exploratory analyses.

### Trial status

Recruitment started on 15 January 2018 and will continue until 31 March 2020. Protocol version 3, 26 September 2019.

## Data Availability

Trial materials can be obtained from the first author. Given the highly personal nature of the study data, participants will be asked for optional consent to sharing their anonymised data with other researchers.

## Abbreviations

AUDIT: Alcohol Use Disorders Identification Test
CACE: Complier-adjusted causal effect analysis
CAPS-5: Clinician Administered PTSD Scale for *DSM-5*
CR: Credibility rating
CSRI: Client Service Receipt Inventory
CTS-R: Cognitive Therapy Rating Scale-Revised
*DSM-5*: *The Diagnostic and Statistical Manual of Mental Disorders, Fifth Edition*
EMDR: Eye-movement desensitisation and reprocessing
EQ-5D-5 L: EuroQol-5 Dimensions-5 Levels
GAD-7: Generalised Anxiety Disorder Scale
GCP: Good Clinical Practice
GSES: General Self Efficacy Scale
IAPT: Improving Access to Psychological Therapies
IES-R: Impact of Event Scale-Revised
ISI: Insomnia Severity Index
ITQ: International Trauma Questionnaire
LEC-5: Life Events Checklist for *DSM-5*
MQ: Trauma Memory Questionnaire
NHS: National Health Service
NICE: National Institute for Health and Care Excellence
NIHR: National Institute of Health Research
non-CTIMP: Clinical trial not involving Investigational Medical Products
PCL-5: PTSD Checklist for *DSM-5*
PCQ: iMTA Productivity Cost Questionnaire
PEQ: IAPT Patient Experience Questionnaire (online version)
PHQ-9: Patient Health Questionnaire
PTCI: Post-Traumatic Cognitions Inventory
PTSD: Post-Traumatic Stress Disorder
QALY: Quality-adjusted life year
QoL: Endicott Quality of Life Scale
RCT: Randomised controlled trial
RIQ: Response to Intrusion Questionnaire
SAE: Serious adverse event
SAPAS: Standardised Assessment of Personality Abbreviated Scale
SBQ: Safety Behaviours Questionnaire
SCID-5: Structured Clinical Interview for *DSM-5*
SCID-5-PD: Structured Clinical Interview for *DSM-5* Personality Disorders
TF-CBT: Trauma-focussed cognitive behavioural therapy
TFPT: Trauma-focussed psychological therapy
TOC: Trial Oversight Committee
TSDQ: Trait-State Dissociation Questionnaire
WAI: Working Alliance Inventory
WHO-5: WHO(Five) Well-Being Index
